# Durable remissions are rare following high dose therapy with autologous stem cell transplantation for adults with "paediatric" bone and soft tissue sarcomas

**DOI:** 10.1186/1477-7800-2-12

**Published:** 2005-05-31

**Authors:** Shriram V Nath, H Miles Prince, Peter FM Choong, Guy C Toner

**Affiliations:** 1 Haematology Service, Peter MacCallum Cancer Centre, St. Andrew's Place, East Melbourne, Australia; 2 Sarcoma Service, Peter MacCallum Cancer Centre, St. Andrew's Place, East Melbourne, Australia; 3 University of Melbourne, Australia

**Keywords:** Ewings sarcoma, Osteosarcoma, Rhabdomyosarcoma, High dose chemotherapy, transplantation

## Abstract

**Background:**

The role of high dose therapy (HDT) with autologous stem cell transplantation (AuSCT) for the treatment of bone and soft tissue sarcomas remains investigational. There are few reports examining this strategy focusing on the adult population.

**Methods:**

We retrospectively reviewed our experience of adult patients undergoing HDT and AuSCT for 'paediatric' sarcomas.

**Results:**

A total of 17 patients (14 male, 3 female) with median age at transplant of 24 years (range 20 – 41) were identified. The diagnosis was Ewings sarcoma/PNET (10), osteosarcoma (5) and rhabdomyosarcoma (2). Status prior to HDT, following conventional-dose chemotherapy +/- surgery +/- radiotherapy, was complete remission (CR) (6), partial remission (PR) (6), stable disease (1) and progressive disease (4). There was no transplant-related mortality. Two patients remain disease free beyond four years and both received HDT as part of their primary therapy (CR1 and PR1) however, the median progression free survival and overall survival following AuSCT for the entire cohort was only 7 months (range: 2–92 months) and 13 months (range: 2 – 92 months), respectively.

**Conclusion:**

HDT and AuSCT infrequently achieves prolonged remissions in adult patients and should only be considered in patients who are in a PR or CR following conventional-dose therapy. Further studies are required to define the role of HDT with AuSCT for adult patients with sarcoma.

## Introduction

The role of high dose chemotherapy (HDT) and autologous stem cell transplantation (AuSCT) for the treatment of patients with sarcoma remains controversial. [1-3]

The most common sarcoma for which HDT and AuSCT has been investigated is the Primitive Neuroectodermal Ectodermal Tumor (PNET) family of tumours which includes Ewing's sarcoma (ES). Indeed, the median age at diagnosis of PNET is14 years with 90% of patients being under the age of 20 years.[4] In adults with PNET, the 5-year overall survival (OS) following conventional-dose therapy is between 38–60% while the progression-free survival (PFS) is 27–59%. [5-7] Conversely, adult and paediatric patients who fail to achieve a complete response following surgery and conventional-dose chemotherapy are considered incurable and those with distant metastases have only 9% – 33% long term survival.[8] With respect to the role of HDT with AuSCT, no randomized studies have been performed and most published studies have included predominantly paediatric patients. [9-16]

Similarly, there is limited data on the role of HDT and AuSCT for osteosarcoma.[17] Again, studies to date have predominantly involved paediatric patients.[1,18,19]

The most common soft tissue sarcoma (STS) in children is rhabdomyosarcoma (RMS). Rare in adults, the disease free survival at 5 years of adult STS (different histologies) is approximately 10%. [20-25] In general, aggressive local therapy consisting of maximum tumor bulk reduction with surgery, with or without radiotherapy, is the cornerstone of initial management and patients with STS who are rendered disease free after surgery have a superior DFS than those who are inoperable.[26] However, many patients with STS are diagnosed with advanced-stage of the disease and/or complete surgical resection of the tumor is not feasible. Moreover, 40 to 60% of patients, even after a primarily curative local therapy, will develop metastases most frequently occurring within 2 to 3 years, and they will ultimately die of the disease.[27] Adult patients with RMS treated with conventional-dose therapy have an even worse prognosis than children.[28] The role of HDT and AuSCT for RMS remains unclear.[11,29]

Given the results with conventional-dose therapy, the role of HDT with AuSCT for sarcomas has largely been explored for patients with relapsed disease or those at high-risk of relapse. Patients with adult STS generally are poorly responsive to conventional-dose chemotherapy, with doxorubicin and ifosfamide being the only available agents showing response rates of greater than 20%. Combination regimens generally do not add efficacy, but do add toxicity.[21,24,27] For both agents, a dose-response relationship has been shown. Despite this theoretical rationale, the benefit of HDT is far from established in paediatric population, let alone for adult patients with sarcomas, with only a few studies reported and with small patient numbers.[9-11,17,18,27,30]

Of the studies reported to date, improved survival rates appear to be achieved for patients who achieve a complete remission (CR) prior to HDT or for those where total resection of tumor is feasible either before or after HDT. [10-12,26,30] Conversely, HDT with AuSCT appears not to improve outcome for PNET patients with metastatic disease at presentation.[13-16,31] Here we report our experience in treating *adult *patients with PNET, osteosarcoma and RMS.

## Materials and methods

A retrospective review of adult patients who underwent HDT with AuSCT for sarcoma since 1992 at our institute was performed. Toxicity was assessed according to the World Health Organisation scale. Survival curves were generated according to the Kaplan-Meier method with OS and PFS calculated from the date of first stem cell infusion.

## Results

A total of 17 patients underwent HDT and AuSCT and their characteristics are presented in Table 1 and included ES (9), PNET (1), osteosarcoma (5) and RMS (2). There were 14 males and 3 females with the median age at time of AuSCT of 24 years (range 20–41 years). HDT was administered as part of initial treatment (8), of which only one was in CR, five in PR, one with progressive disease (ProD) and one with stable disease (SD); in first relapse (7), of which four were in CR, one in PR and two with ProD; fourth relapse (1) who was in CR. Disease status immediately prior to HDT (following conventional-dose chemotherapy and/or surgery and/or radiotherapy) was CR (6), partial remission (PR) (6), stable disease (SD) (1) and ProD (4). The source of autologous stem cells was peripheral blood for 15 patients and bone marrow for two patients. Eleven patients underwent a planned tandem transplant.

**Table 1:** Summary of Patient Characteristics and Outcome

**No**	**age at AuSCT**	**gender**	**diagnosis**	**surgery pre AuSCT**	**disease site at HDT**	**status at AuSCT**	**time between diagnosis and AuSCT (months)**	Regimen*	**status post AuSCT**	**surgery post AuSCT**	**TTP (months)**	**status**	**time to last follow up/death (months)**
1	39	M	ES	Y	Rib	CR1	11	e	CR1	N	NA	A	57
2	20	F	ES	Y	Lung	CR2	38	c	CR2	N	9	D	13
3	24	F	ES	Y	Brain	CR2	37	f	CR2	N	NA	A	3
4	32	M	ES	N	Pelvis	PR1	4	g, b	PR1	N	32	D	64
5	20	M	ES	N	Scapula Clavicle	PR1	6	g, b	PR1	N	4	D	5
6	24	M	PNET	Y	Brain	PR2	52	f, b	SD2	N	NA	A	4
7	41	M	ES	Y	Tibia	PR1	8	a, a	PROG1	N	3	D	4
8	24	M	ES	N	Lung Bones	ProD2	40	c	PR2	Y	5**	D	5
9	27	M	ES	N	Spine	ProD1	7	d	PROG1	N	5	D	2
10	26	M	ES	Y	Spine	SD1	23	g, b	SD1	N	5	D	6
11	25	M	OS	Y	Lung	CR2	17	a, a	CR2	N	7	D	15
12	21	M	OS	Y	Lung	CR4	84	a, b	CR4	N	NA	A	12
13	24	M	OS	Y	Node, Rib, Scapula	CR2	24	a, a	CR2	N	6	D	25
14	22	M	OS	Y	Lung	ProD2	34	a	PR2	N	3	D	5
15	28	M	OS	Y	Lung	ProD3	32	a, b	PR3	N	10	D	13
16	21	M	RMS	Y	bladder	PR1	13	g, h	CR1	N	NA	A	92
17	34	F	RMS	Y	face	PR1	5	g, i	CR1	N	NA	D	7

AuSCT = autologous stem cells transplant, TTP = time to progression,*See text for details of conditioning regimen, ES = Ewing's Sarcoma, PNET = Peripheral Neuroectodermal Tumor, OS = osteosarcoma, RMS = rhabdomyosarcoma, CR = Complete Remission, PR = Partial Remission, ProD = Progressive disease, SD = stable disease, NA = Not Applicable, A = alive, D = dead, **Travelled back to his native country and died (cause unknown but assumed to be disease-related).

HDT regimens were selected depending on prior therapy or involvement in specific clinical trials. Patients with osteosarcoma received carboplatin (700 mg/m^2^) and etoposide (750 mg/m^2^)^a^. Four patients subsequently received a second HDT and AuSCT with the same regimen (2) or melphalan (180 mg/m^2^) and etoposide (60 mg/kg)^b ^(2). Patients with PNET/ES received a single course of melphalan (180 mg/m^2^)^c^(2) or radionuclide Samarium^153 ^EDTMP^d^(1) or carboplatin (700 mg/m^2^) and etoposide (750 mg/m^2^)^e^(1) or carboplatin (AUC = 21), thiotepa (900 mg/m^2^) and etoposide (750 mg/m^2^)^f ^(1) or tandem cycles with ifosfamide (12 g/m^2^), carboplatin (AUC = 20) and etoposide (60 mg/kg)^g ^followed by and melphalan (180 mg/m^2^) and etoposide (60 mg/kg)^b ^(3);or tandem cycles with two cycles of carboplatin (700 mg/m^2^) and etoposide (750 mg/m2)^a ^(1); or tandem cycles with carboplatin (AUC = 21), thiotepa (900 mg/m^2^) and etoposide (750 mg/m^2^)^f ^followed by melphalan (180 mg/m^2^) and etoposide (60 mg/kg)^b ^(1). The two patients with RMS were treated with tandem cycles of HDT with AuSCT consisting of ifosfamide (12 g/m^2^), carboplatin (AUC = 20) and etoposide (60 mg/kg)^g ^followed by mitoxantrone (48 mg/m^2^), carboplatin (AUC = 20) and etoposide (60 mg/kg)^h ^(1) or ifosfamide (12 g/m^2^), carboplatin (AUC = 16) mitoxantrone (64 mg/m^2^)^i ^(1). (Superscripted letters- indicating conditioning regimens used in Table 1)

All patients experienced WHO grade 4 leukopenia, neutropenia and thrombocytopenia after the HDT. The other WHO grade 3–4 toxicities were mucositis (3), pulmonary haemorrhage (1), ileitis (2) and haemorrhagic cystitis (1). There were no transplant-related deaths.

Median follow up for all surviving patients was 12 months. Of patients in CR at the time of HDT (6), none progressed during HDT and three of these patients have subsequently relapsed and died with a median PFS of nine months (range: 3–57 months). In patients entering HDT in PR (6), two achieved CR after AuSCT – one (pt 17) subsequently died of bilateral frontal haemorrhage at 7 months (not disease related), two achieved further reduction in tumour without achieving CR, one had SD and one had ProD. All of the patients who had either ProD (4) or SD (1) at the time of HDT subsequently progressed and died.

**Figure 1:**
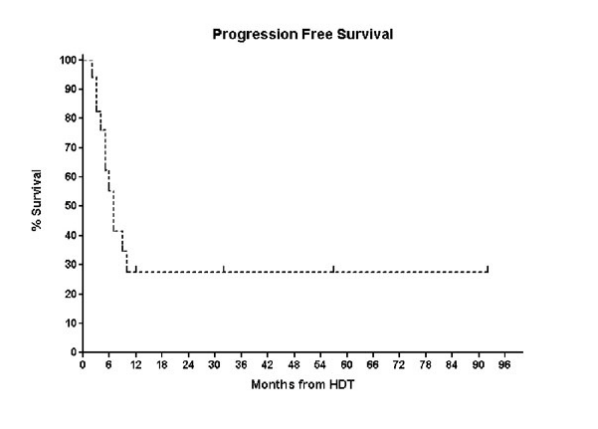
Kaplan-Meier Curve of Progression free Survival for the entire cohort.

**Figure 2:**
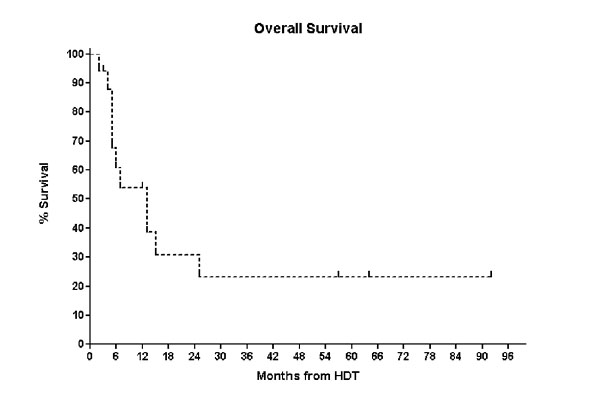
Kaplan-Meier Curve of Progression free Survival for the entire cohort.

Of the eight patients who were in CR following HDT, three relapsed and died, four remain alive and disease free (3 – 92 months). Two patients remain disease free beyond four years (Pt 1 and Pt 16) and both received HDT as part of their primary therapy (CR1 and PR1) however, the median PFS and OS following AuSCT for the entire cohort was only 7 months (range: 2–92 months) and 13 months (range: 2 – 92 months), respectively. (Figures 1 &2).

## Discussion

Given the relative chemotherapy-sensitivity of paediatric sarcomas and the poor prognosis with conventional-dose therapy for high-risk or relapsed disease, HDT with AuSCT has been investigated. Due to the rarity of these tumours, most studies have been retrospective, relatively small and have examined heterogeneous patient populations. Thus, despite some promising results demonstrating longer than expected survival, the benefits of this strategy remains uncertain.[27]

There is currently little data on the role of HDT for "paediatric" sarcomas in the *adult *sarcoma population. Our results in this group are disappointing. The median OS for the entire population was only thirteen months (range: 2–92 months) with a median PFS of seven months (range: 2–92 months) with only two patients remaining disease free beyond four years. Meyer et al. demonstrated that consolidation with high dose chemo-radiotherapy followed by stem cell support failed to improve the probability of EFS in a cohort of patients with newly diagnosed metastatic Ewings Sarcoma.[16]

With respect to osteosarcoma, we utilized a tandem transplant approach similar to that of the Italian sarcoma group (two courses of high dose carboplatin and etoposide) and they have recently demonstrated in 32 patients, with a median age of 15 years, a transplant-related mortality of 3.1% and a progression rate of 84% with a three-year OS and DFS of 20% and 12%, respectively.[24]

Comparative results of other published trials in STS, most of which include a paediatric population are presented in Table 2. Of note, Blay et al. reported an eight percent five year survival in adult patients with metastatic or locally advanced irresectable STS [12] and Bokenmeyer et al[32] reported results similar to ours in a cohort of 18 adult patients with STS.

**Table 2:** Summary of High Dose Chemotherapy Studies in Soft Tissue Sarcomas

**Author**	**Number of Pts**	**Median Age (Range)**	**R.F.S (months)**	**O.S. (months)**
Kasper ^29^	27	30.6 (13–59)	12	16.5
Blay ^12^	30	34 (17–57)	7	19
Samuel ^34^	23	not stated	-	5.1
Dumontet^35^	22	16 (3–45)	15	19
Bokemeyer^32^	18	45 (25–37)	8	13
Kang^36^	24	24 (11–53)	6	10
Schlemmer^37^	55	not stated	-	23

The European Organization for Research and Treatment of Cancer demonstrated that predictors for improved survival for STS and bone sarcomas following HDT were performance status, female gender, grade I tumors and the achievement of a CR after first line treatment.[33] In our study there was no clear predictors of durable remissions; three of the six patients who underwent HDT in CR relapsed, with a median PFS of nine months. (Range 3–57 months). Similarly, four of the eight who were in CR following HDT relapsed and died. It is of interest that one patient remains disease free at 12 months undergoing HDT and AuSCT in fourth CR.

## Conclusion

Our results demonstrate that HDT and AuSCT infrequently achieves prolonged remissions in adult patients and only prospective studies will definitely determine the place of HDT in this group. Our data support that contention that HDT should only be considered in patients who are in a PR or CR following conventional-dose therapy.

## Abbreviations

AuSCT Autologous stem cell transplantation

CR Complete remission

ES Ewing's sarcoma

HDT High dose therapy

OS Overall survival

PR Partial remission

PNET Primitive Neuroectodermal Ectodermal Tumor

PFS Progression free survival

ProD Progressive Disease

RMS Rhabdomyosarcoma

STS Soft tissue sarcoma

SD Stable disease

## Authors' contributions

SVN, HMP, PFMC, GCT conceived the study and participated in its design and helped draft the manuscript. Authors read and approved the final manuscript.
